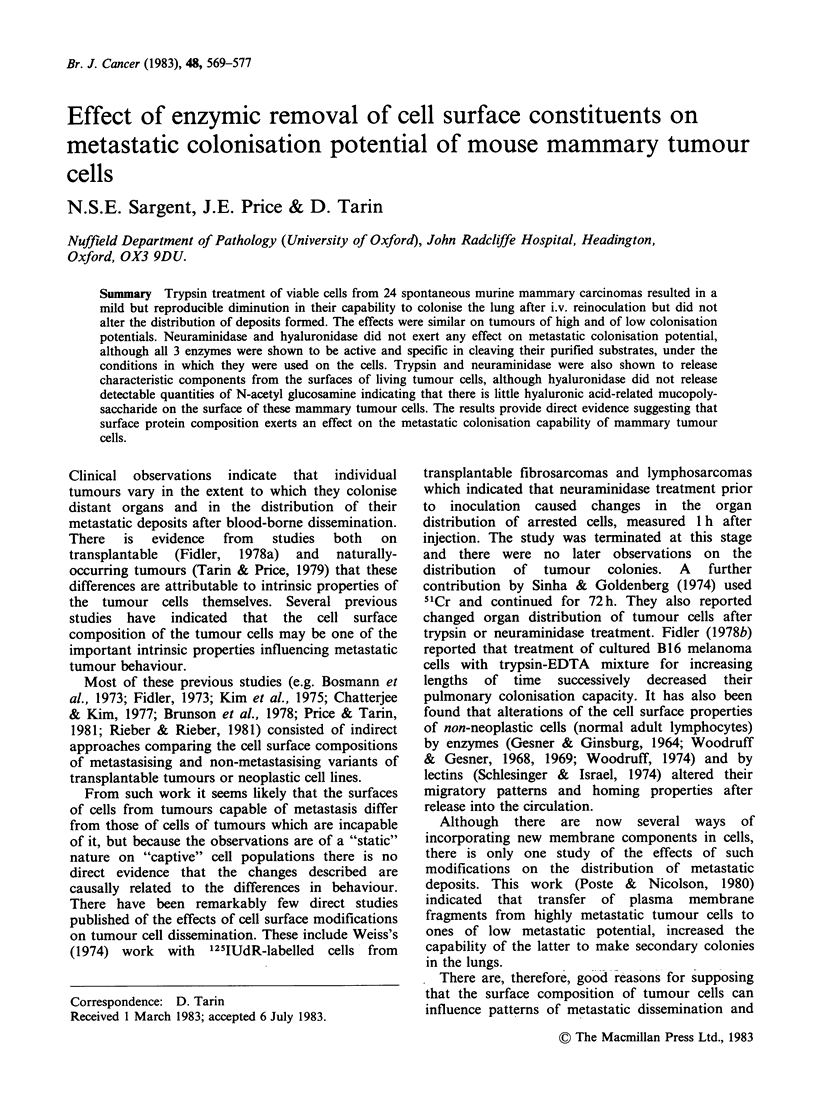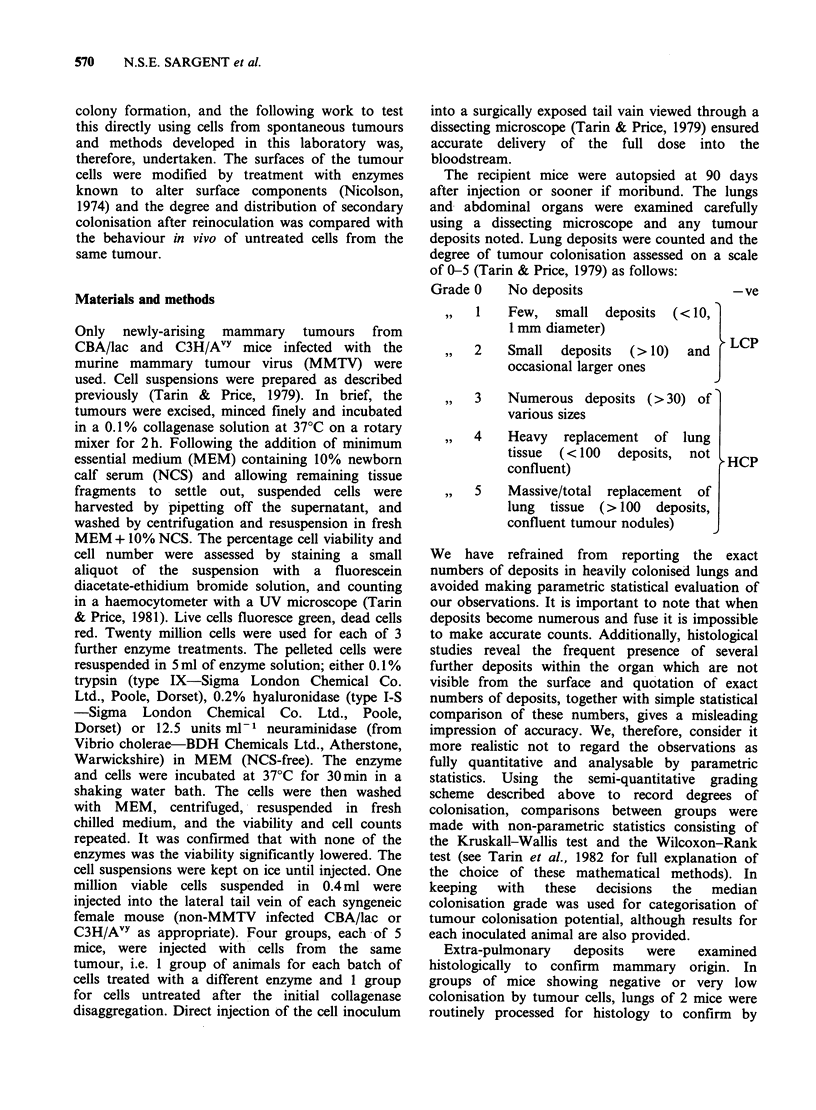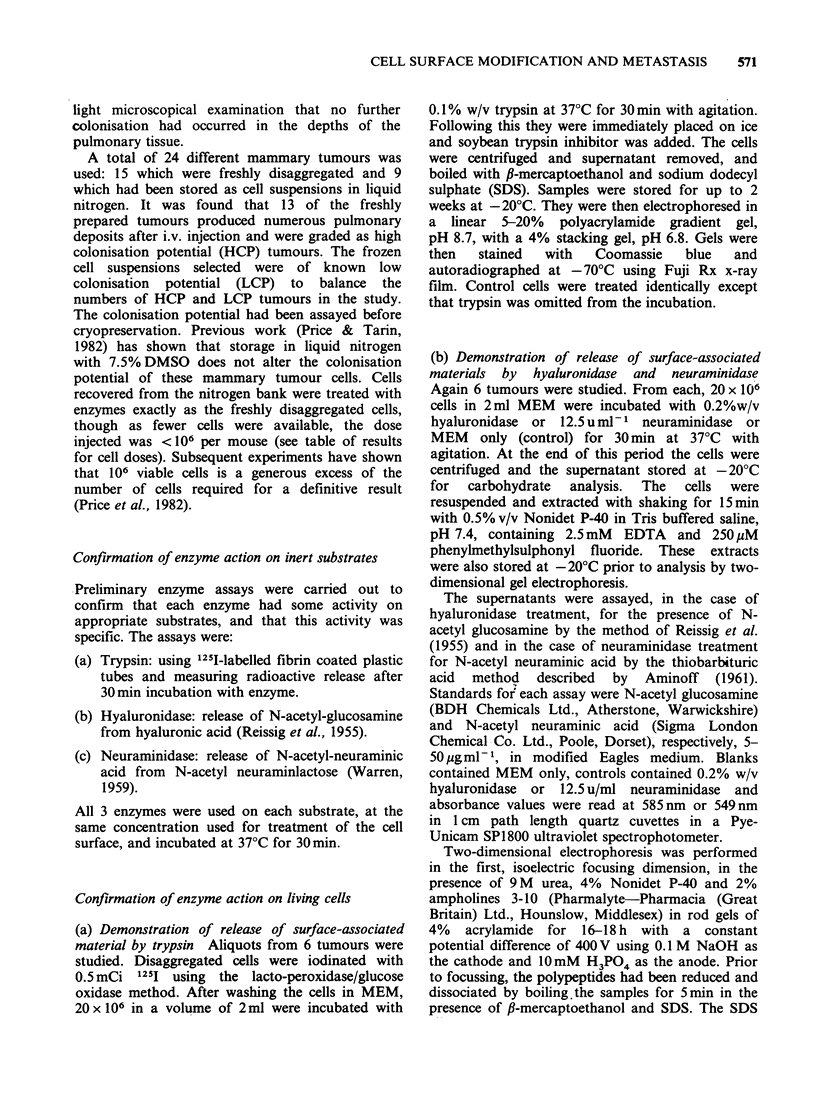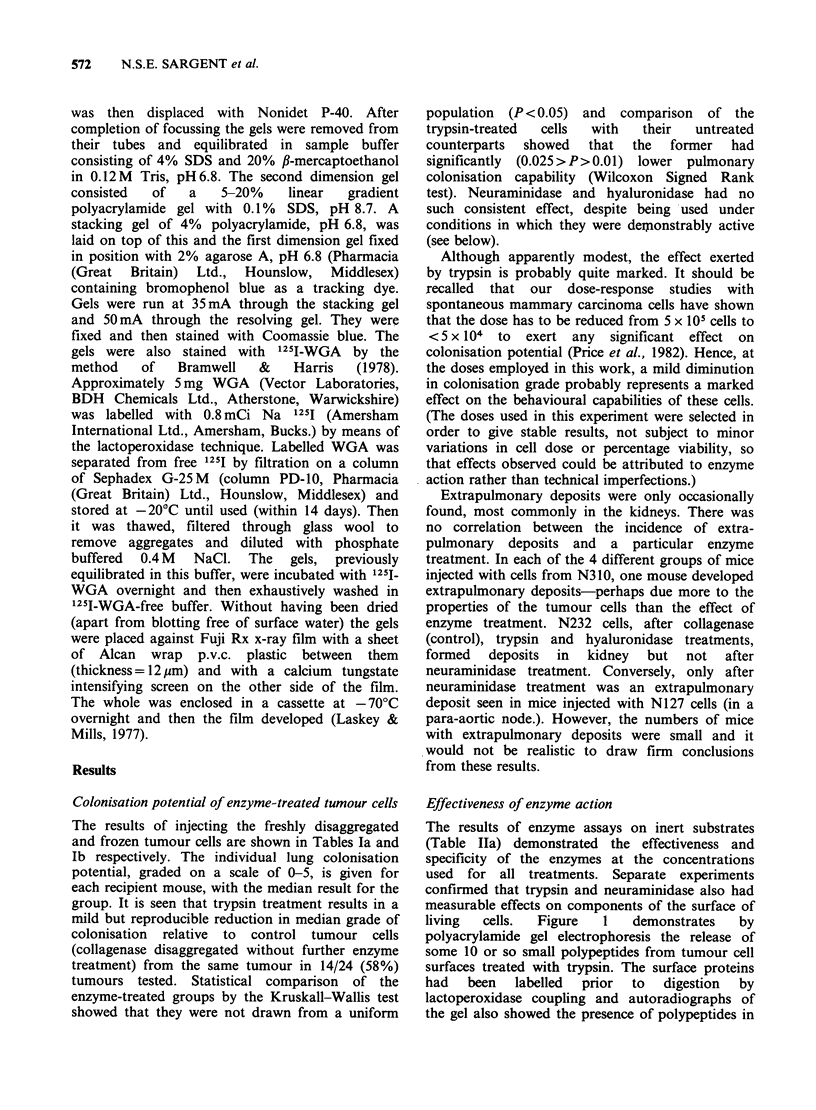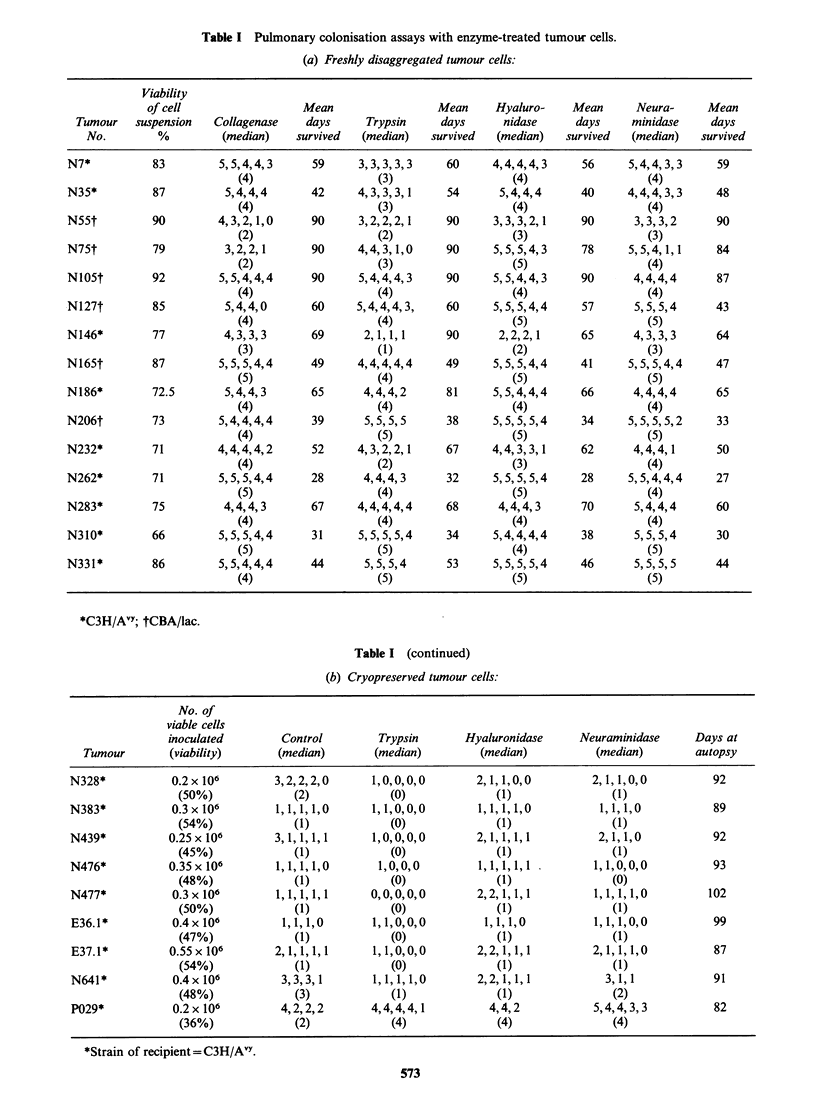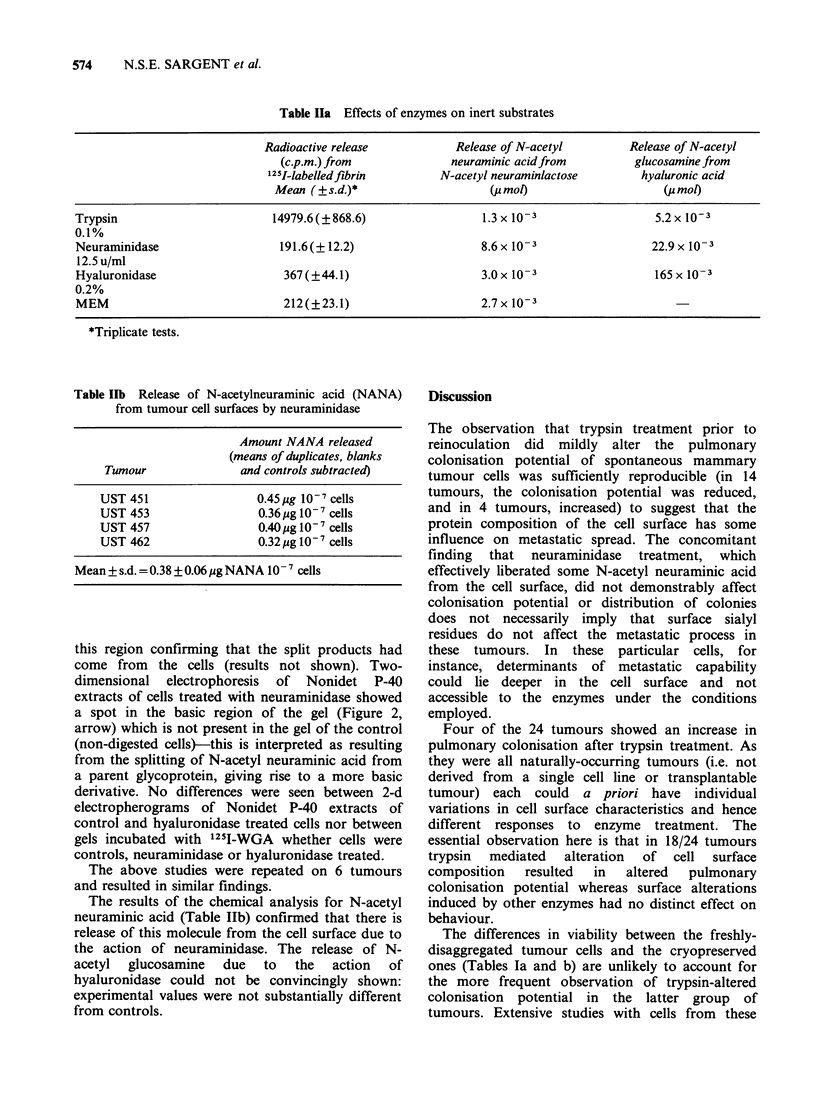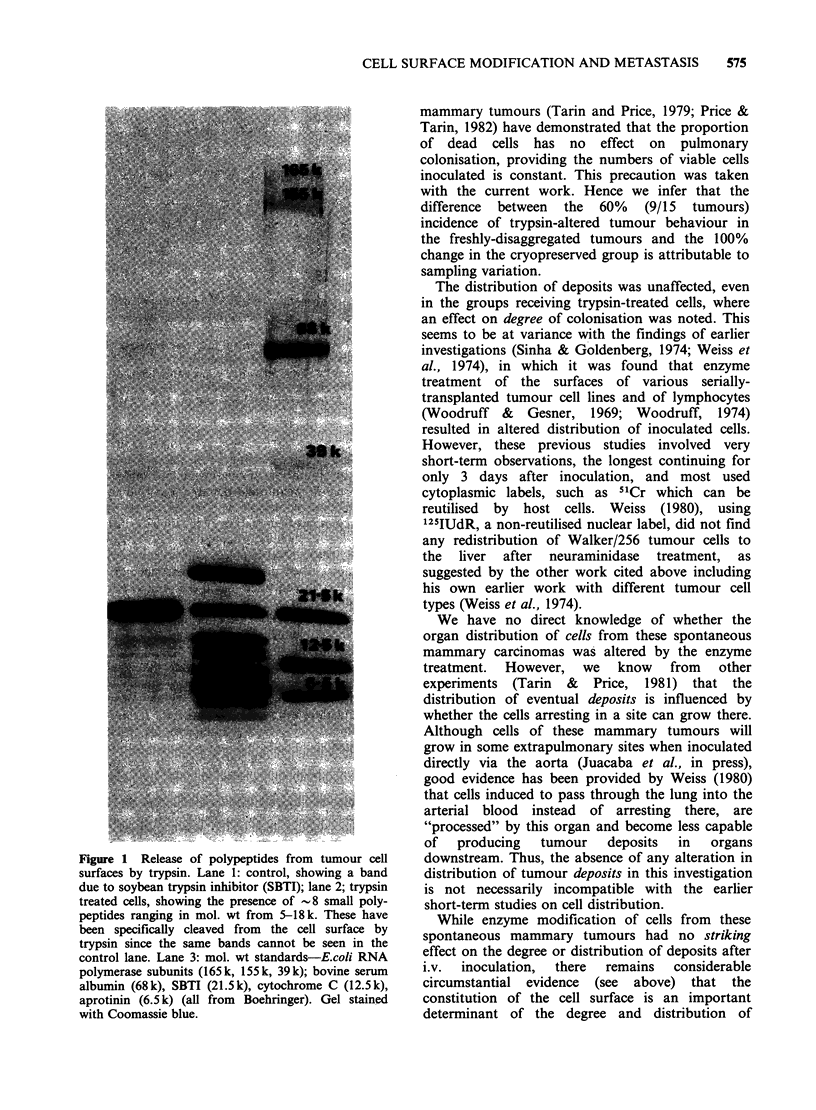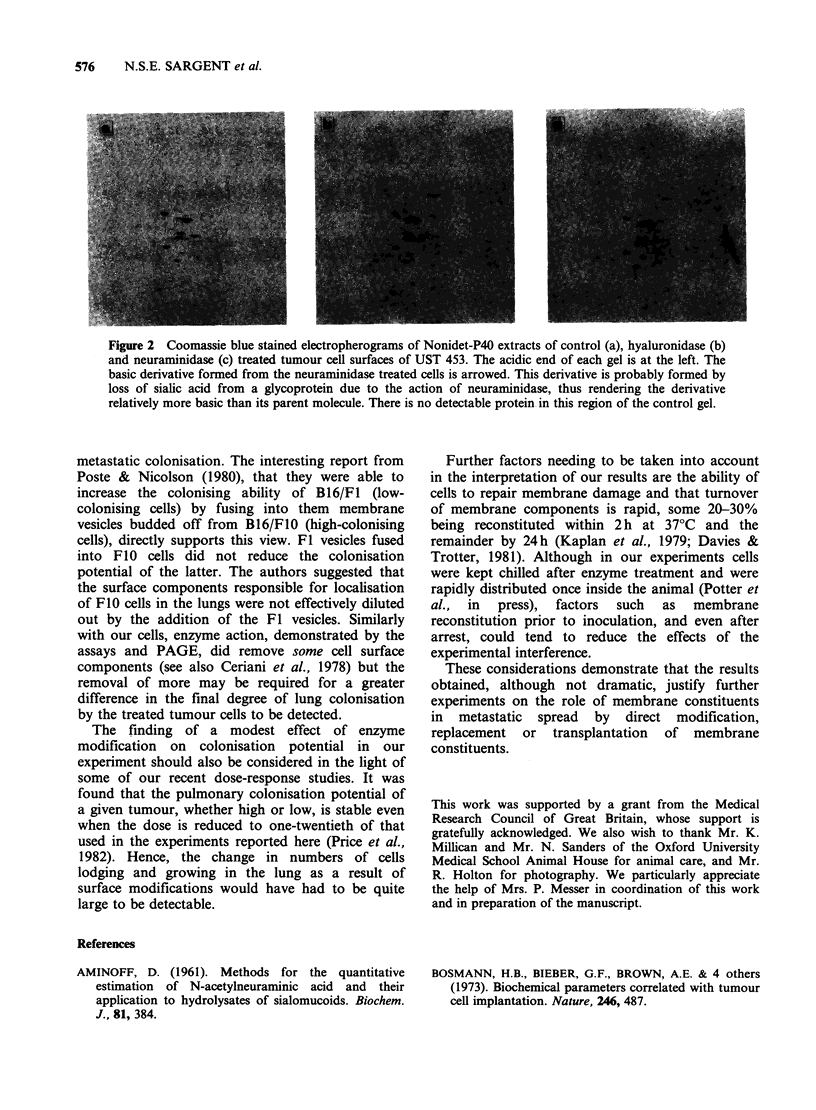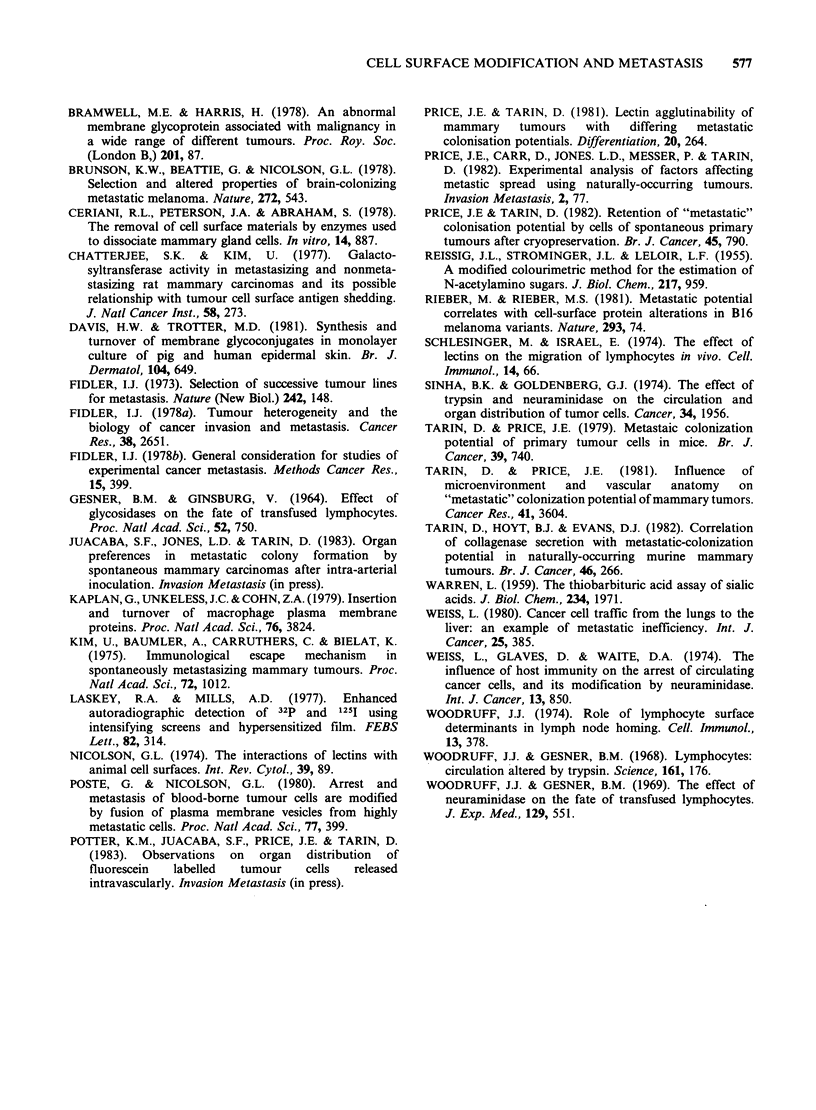# Effect of enzymic removal of cell surface constituents on metastatic colonisation potential of mouse mammary tumour cells.

**DOI:** 10.1038/bjc.1983.230

**Published:** 1983-10

**Authors:** N. S. Sargent, J. E. Price, D. Tarin

## Abstract

**Images:**


*Br. J. Cancer* (1983), **48**, 569–577

### Effect of enzymic removal of cell surface constituents on metastatic colonisation potential of mouse mammary tumour cells

N.S.E. Sargent, J.E. Price & D. Tarin

*Nuffield Department of Pathology (University of Oxford), John Radcliffe Hospital, Headington, Oxford, OX3 9DU.*

**Summary** Trypsin treatment of viable cells from 24 spontaneous murine mammary carcinomas resulted in a mild but reproducible diminution in their capability to colonise the lung after i.v. reinoculation but did not alter the distribution of deposits formed. The effects were similar on tumours of high and of low colonisation potentials. Neuraminidase and hyaluronidase did not exert any effect on metastatic colonisation potential, although all 3 enzymes were shown to be active and specific in cleaving their purified substrates, under the conditions in which they were used on the cells. Trypsin and neuraminidase were also shown to release characteristic components from the surfaces of living tumour cells, although hyaluronidase did not release detectable quantities of N-acetyl glucosamine indicating that there is little hyaluronic acid-related mucopolysaccharide on the surface of these mammary tumour cells. The results provide direct evidence suggesting that surface protein composition exerts an effect on the metastatic colonisation capability of mammary tumour cells.

Clinical observations indicate that individual tumours vary in the extent to which they colonise distant organs and in the distribution of their metastatic deposits after blood-borne dissemination. There is evidence from studies both on transplantable (Fidler, 1978a) and naturally-occurring tumours (Tarin & Price, 1979) that these differences are attributable to intrinsic properties of the tumour cells themselves. Several previous studies have indicated that the cell surface composition of the tumour cells may be one of the important intrinsic properties influencing metastatic tumour behaviour.

Most of these previous studies (e.g. Bosmann *et al.*, 1973; Fidler, 1973; Kim *et al.*, 1975; Chatterjee & Kim, 1977; Brunson *et al.*, 1978; Price & Tarin, 1981; Rieber & Rieber, 1981) consisted of indirect approaches comparing the cell surface compositions of metastasising and non-metastasising variants of transplantable tumours or neoplastic cell lines.

From such work it seems likely that the surfaces of cells from tumours capable of metastasis differ from those of cells of tumours which are incapable of it, but because the observations are of a "static" nature on "captive" cell populations there is no direct evidence that the changes described are causally related to the differences in behaviour. There have been remarkably few direct studies published of the effects of cell surface modifications on tumour cell dissemination. These include Weiss's (1974) work with $^{125}$IUdR-labelled cells from transplantable fibrosarcomas and lymphosarcomas which indicated that neuraminidase treatment prior to inoculation caused changes in the organ distribution of arrested cells, measured 1 h after injection. The study was terminated at this stage and there were no later observations on the distribution of tumour colonies. A further contribution by Sinha & Goldenberg (1974) used $^{51}$Cr and continued for 72 h. They also reported changed organ distribution of tumour cells after trypsin or neuraminidase treatment. Fidler (1978*b*) reported that treatment of cultured B16 melanoma cells with trypsin-EDTA mixture for increasing lengths of time successively decreased their pulmonary colonisation capacity. It has also been found that alterations of the cell surface properties of *non*-neoplastic cells (normal adult lymphocytes) by enzymes (Gesner & Ginsburg, 1964; Woodruff & Gesner, 1968, 1969; Woodruff, 1974) and by lectins (Schlesinger & Israel, 1974) altered their migratory patterns and homing properties after release into the circulation.

Although there are now several ways of incorporating new membrane components in cells, there is only one study of the effects of such modifications on the distribution of metastatic deposits. This work (Poste & Nicolson, 1980) indicated that transfer of plasma membrane fragments from highly metastatic tumour cells to ones of low metastatic potential, increased the capability of the latter to make secondary colonies in the lungs.

There are, therefore, good reasons for supposing that the surface composition of tumour cells can influence patterns of metastatic dissemination and

Correspondence: D. Tarin
Received 1 March 1983; accepted 6 July 1983.

© The Macmillan Press Ltd., 1983

colony formation, and the following work to test this directly using cells from spontaneous tumours and methods developed in this laboratory was, therefore, undertaken. The surfaces of the tumour cells were modified by treatment with enzymes known to alter surface components (Nicolson, 1974) and the degree and distribution of secondary colonisation after reinoculation was compared with the behaviour *in vivo* of untreated cells from the same tumour.

### Materials and methods

Only newly-arising mammary tumours from CBA/lac and C3H/$A^{vy}$ mice infected with the murine mammary tumour virus (MMTV) were used. Cell suspensions were prepared as described previously (Tarin & Price, 1979). In brief, the tumours were excised, minced finely and incubated in a 0.1% collagenase solution at 37°C on a rotary mixer for 2 h. Following the addition of minimum essential medium (MEM) containing 10% newborn calf serum (NCS) and allowing remaining tissue fragments to settle out, suspended cells were harvested by pipetting off the supernatant, and washed by centrifugation and resuspension in fresh MEM + 10% NCS. The percentage cell viability and cell number were assessed by staining a small aliquot of the suspension with a fluorescein diacetate-ethidium bromide solution, and counting in a haemocytometer with a UV microscope (Tarin & Price, 1981). Live cells fluoresce green, dead cells red. Twenty million cells were used for each of 3 further enzyme treatments. The pelleted cells were resuspended in 5 ml of enzyme solution; either 0.1% trypsin (type IX—Sigma London Chemical Co. Ltd., Poole, Dorset), 0.2% hyaluronidase (type I-S —Sigma London Chemical Co. Ltd., Poole, Dorset) or 12.5 units $ml^{-1}$ neuraminidase (from Vibrio cholerae—BDH Chemicals Ltd., Atherstone, Warwickshire) in MEM (NCS-free). The enzyme and cells were incubated at 37°C for 30 min in a shaking water bath. The cells were then washed with MEM, centrifuged, resuspended in fresh chilled medium, and the viability and cell counts repeated. It was confirmed that with none of the enzymes was the viability significantly lowered. The cell suspensions were kept on ice until injected. One million viable cells suspended in 0.4 ml were injected into the lateral tail vein of each syngeneic female mouse (non-MMTV infected CBA/lac or C3H/$A^{vy}$ as appropriate). Four groups, each of 5 mice, were injected with cells from the same tumour, i.e. 1 group of animals for each batch of cells treated with a different enzyme and 1 group for cells untreated after the initial collagenase disaggregation. Direct injection of the cell inoculum into a surgically exposed tail vain viewed through a dissecting microscope (Tarin & Price, 1979) ensured accurate delivery of the full dose into the bloodstream.

The recipient mice were autopsied at 90 days after injection or sooner if moribund. The lungs and abdominal organs were examined carefully using a dissecting microscope and any tumour deposits noted. Lung deposits were counted and the degree of tumour colonisation assessed on a scale of 0–5 (Tarin & Price, 1979) as follows:

| | | |
|---|---|---|
| Grade 0 | No deposits | −ve |
| „ 1 | Few, small deposits (<10, 1 mm diameter) | LCP |
| „ 2 | Small deposits (>10) and occasional larger ones | |
| „ 3 | Numerous deposits (>30) of various sizes | HCP |
| „ 4 | Heavy replacement of lung tissue (<100 deposits, not confluent) | |
| „ 5 | Massive/total replacement of lung tissue (>100 deposits, confluent tumour nodules) | |

We have refrained from reporting the exact numbers of deposits in heavily colonised lungs and avoided making parametric statistical evaluation of our observations. It is important to note that when deposits become numerous and fuse it is impossible to make accurate counts. Additionally, histological studies reveal the frequent presence of several further deposits within the organ which are not visible from the surface and quotation of exact numbers of deposits, together with simple statistical comparison of these numbers, gives a misleading impression of accuracy. We, therefore, consider it more realistic not to regard the observations as fully quantitative and analysable by parametric statistics. Using the semi-quantitative grading scheme described above to record degrees of colonisation, comparisons between groups were made with non-parametric statistics consisting of the Kruskall–Wallis test and the Wilcoxon–Rank test (see Tarin *et al.*, 1982 for full explanation of the choice of these mathematical methods). In keeping with these decisions the median colonisation grade was used for categorisation of tumour colonisation potential, although results for each inoculated animal are also provided.

Extra-pulmonary deposits were examined histologically to confirm mammary origin. In groups of mice showing negative or very low colonisation by tumour cells, lungs of 2 mice were routinely processed for histology to confirm by light microscopical examination that no further colonisation had occurred in the depths of the pulmonary tissue.

A total of 24 different mammary tumours was used: 15 which were freshly disaggregated and 9 which had been stored as cell suspensions in liquid nitrogen. It was found that 13 of the freshly prepared tumours produced numerous pulmonary deposits after i.v. injection and were graded as high colonisation potential (HCP) tumours. The frozen cell suspensions selected were of known low colonisation potential (LCP) to balance the numbers of HCP and LCP tumours in the study. The colonisation potential had been assayed before cryopreservation. Previous work (Price & Tarin, 1982) has shown that storage in liquid nitrogen with 7.5% DMSO does not alter the colonisation potential of these mammary tumour cells. Cells recovered from the nitrogen bank were treated with enzymes exactly as the freshly disaggregated cells, though as fewer cells were available, the dose injected was $<10^6$ per mouse (see table of results for cell doses). Subsequent experiments have shown that $10^6$ viable cells is a generous excess of the number of cells required for a definitive result (Price *et al.*, 1982).

*Confirmation of enzyme action on inert substrates*

Preliminary enzyme assays were carried out to confirm that each enzyme had some activity on appropriate substrates, and that this activity was specific. The assays were:

(a) Trypsin: using $^{125}$I-labelled fibrin coated plastic tubes and measuring radioactive release after 30 min incubation with enzyme.

(b) Hyaluronidase: release of N-acetyl-glucosamine from hyaluronic acid (Reissig *et al.*, 1955).

(c) Neuraminidase: release of N-acetyl-neuraminic acid from N-acetyl neuraminlactose (Warren, 1959).

All 3 enzymes were used on each substrate, at the same concentration used for treatment of the cell surface, and incubated at 37°C for 30 min.

*Confirmation of enzyme action on living cells*

(a) *Demonstration of release of surface-associated material by trypsin* Aliquots from 6 tumours were studied. Disaggregated cells were iodinated with 0.5 mCi $^{125}$I using the lacto-peroxidase/glucose oxidase method. After washing the cells in MEM, $20\times10^6$ in a volume of 2 ml were incubated with 0.1% w/v trypsin at 37°C for 30 min with agitation. Following this they were immediately placed on ice and soybean trypsin inhibitor was added. The cells were centrifuged and supernatant removed, and boiled with $\beta$-mercaptoethanol and sodium dodecyl sulphate (SDS). Samples were stored for up to 2 weeks at −20°C. They were then electrophoresed in a linear 5–20% polyacrylamide gradient gel, pH 8.7, with a 4% stacking gel, pH 6.8. Gels were then stained with Coomassie blue and autoradiographed at −70°C using Fuji Rx x-ray film. Control cells were treated identically except that trypsin was omitted from the incubation.

(b) *Demonstration of release of surface-associated materials by hyaluronidase and neuraminidase* Again 6 tumours were studied. From each, $20\times10^6$ cells in 2 ml MEM were incubated with 0.2%w/v hyaluronidase or 12.5 u ml$^{-1}$ neuraminidase or MEM only (control) for 30 min at 37°C with agitation. At the end of this period the cells were centrifuged and the supernatant stored at −20°C for carbohydrate analysis. The cells were resuspended and extracted with shaking for 15 min with 0.5% v/v Nonidet P-40 in Tris buffered saline, pH 7.4, containing 2.5 mM EDTA and 250 $\mu$M phenylmethylsulphonyl fluoride. These extracts were also stored at −20°C prior to analysis by two-dimensional gel electrophoresis.

The supernatants were assayed, in the case of hyaluronidase treatment, for the presence of N-acetyl glucosamine by the method of Reissig *et al.* (1955) and in the case of neuraminidase treatment for N-acetyl neuraminic acid by the thiobarbituric acid method described by Aminoff (1961). Standards for each assay were N-acetyl glucosamine (BDH Chemicals Ltd., Atherstone, Warwickshire) and N-acetyl neuraminic acid (Sigma London Chemical Co. Ltd., Poole, Dorset), respectively, 5–50 $\mu$g ml$^{-1}$, in modified Eagles medium. Blanks contained MEM only, controls contained 0.2% w/v hyaluronidase or 12.5 u/ml neuraminidase and absorbance values were read at 585 nm or 549 nm in 1 cm path length quartz cuvettes in a Pye-Unicam SP1800 ultraviolet spectrophotometer.

Two-dimensional electrophoresis was performed in the first, isoelectric focusing dimension, in the presence of 9 M urea, 4% Nonidet P-40 and 2% ampholines 3-10 (Pharmalyte—Pharmacia (Great Britain) Ltd., Hounslow, Middlesex) in rod gels of 4% acrylamide for 16–18 h with a constant potential difference of 400 V using 0.1 M NaOH as the cathode and 10 mM $H_3PO_4$ as the anode. Prior to focussing, the polypeptides had been reduced and dissociated by boiling the samples for 5 min in the presence of $\beta$-mercaptoethanol and SDS. The SDS

was then displaced with Nonidet P-40. After completion of focussing the gels were removed from their tubes and equilibrated in sample buffer consisting of 4% SDS and 20% $\beta$-mercaptoethanol in 0.12 M Tris, pH 6.8. The second dimension gel consisted of a 5–20% linear gradient polyacrylamide gel with 0.1% SDS, pH 8.7. A stacking gel of 4% polyacrylamide, pH 6.8, was laid on top of this and the first dimension gel fixed in position with 2% agarose A, pH 6.8 (Pharmacia (Great Britain) Ltd., Hounslow, Middlesex) containing bromophenol blue as a tracking dye. Gels were run at 35 mA through the stacking gel and 50 mA through the resolving gel. They were fixed and then stained with Coomassie blue. The gels were also stained with $^{125}$I-WGA by the method of Bramwell & Harris (1978). Approximately 5 mg WGA (Vector Laboratories, BDH Chemicals Ltd., Atherstone, Warwickshire) was labelled with 0.8 mCi Na $^{125}$I (Amersham International Ltd., Amersham, Bucks.) by means of the lactoperoxidase technique. Labelled WGA was separated from free $^{125}$I by filtration on a column of Sephadex G-25 M (column PD-10, Pharmacia (Great Britain) Ltd., Hounslow, Middlesex) and stored at $-20$°C until used (within 14 days). Then it was thawed, filtered through glass wool to remove aggregates and diluted with phosphate buffered 0.4 M NaCl. The gels, previously equilibrated in this buffer, were incubated with $^{125}$I-WGA overnight and then exhaustively washed in $^{125}$I-WGA-free buffer. Without having been dried (apart from blotting free of surface water) the gels were placed against Fuji Rx x-ray film with a sheet of Alcan wrap p.v.c. plastic between them (thickness = 12 $\mu$m) and with a calcium tungstate intensifying screen on the other side of the film. The whole was enclosed in a cassette at $-70$°C overnight and then the film developed (Laskey & Mills, 1977).

### Results

### *Colonisation potential of enzyme-treated tumour cells*

The results of injecting the freshly disaggregated and frozen tumour cells are shown in Tables Ia and Ib respectively. The individual lung colonisation potential, graded on a scale of 0–5, is given for each recipient mouse, with the median result for the group. It is seen that trypsin treatment results in a mild but reproducible reduction in median grade of colonisation relative to control tumour cells (collagenase disaggregated without further enzyme treatment) from the same tumour in 14/24 (58%) tumours tested. Statistical comparison of the enzyme-treated groups by the Kruskall–Wallis test showed that they were not drawn from a uniform population ($P<0.05$) and comparison of the trypsin-treated cells with their untreated counterparts showed that the former had significantly ($0.025>P>0.01$) lower pulmonary colonisation capability (Wilcoxon Signed Rank test). Neuraminidase and hyaluronidase had no such consistent effect, despite being used under conditions in which they were demonstrably active (see below).

Although apparently modest, the effect exerted by trypsin is probably quite marked. It should be recalled that our dose-response studies with spontaneous mammary carcinoma cells have shown that the dose has to be reduced from $5\times10^5$ cells to $<5\times10^4$ to exert any significant effect on colonisation potential (Price *et al.*, 1982). Hence, at the doses employed in this work, a mild diminution in colonisation grade probably represents a marked effect on the behavioural capabilities of these cells. (The doses used in this experiment were selected in order to give stable results, not subject to minor variations in cell dose or percentage viability, so that effects observed could be attributed to enzyme action rather than technical imperfections.)

Extrapulmonary deposits were only occasionally found, most commonly in the kidneys. There was no correlation between the incidence of extrapulmonary deposits and a particular enzyme treatment. In each of the 4 different groups of mice injected with cells from N310, one mouse developed extrapulmonary deposits—perhaps due more to the properties of the tumour cells than the effect of enzyme treatment. N232 cells, after collagenase (control), trypsin and hyaluronidase treatments, formed deposits in kidney but not after neuraminidase treatment. Conversely, only after neuraminidase treatment was an extrapulmonary deposit seen in mice injected with N127 cells (in a para-aortic node.). However, the numbers of mice with extrapulmonary deposits were small and it would not be realistic to draw firm conclusions from these results.

### *Effectiveness of enzyme action*

The results of enzyme assays on inert substrates (Table IIa) demonstrated the effectiveness and specificity of the enzymes at the concentrations used for all treatments. Separate experiments confirmed that trypsin and neuraminidase also had measurable effects on components of the surface of living cells. Figure 1 demonstrates by polyacrylamide gel electrophoresis the release of some 10 or so small polypeptides from tumour cell surfaces treated with trypsin. The surface proteins had been labelled prior to digestion by lactoperoxidase coupling and autoradiographs of the gel also showed the presence of polypeptides in

**Table I** Pulmonary colonisation assays with enzyme-treated tumour cells.

(*a*) *Freshly disaggregated tumour cells:*

| *Tumour No.* | *Viability of cell suspension %* | *Collagenase (median)* | *Mean days survived* | *Trypsin (median)* | *Mean days survived* | *Hyaluro-nidase (median)* | *Mean days survived* | *Neura-minidase (median)* | *Mean days survived* |
|---|---|---|---|---|---|---|---|---|---|
| N7* | 83 | 5,5,4,4,3 (4) | 59 | 3,3,3,3,3 (3) | 60 | 4,4,4,4,3 (4) | 56 | 5,4,4,3,3 (4) | 59 |
| N35* | 87 | 5,4,4,4 (4) | 42 | 4,3,3,3,1 (3) | 54 | 5,4,4,4 (4) | 40 | 4,4,4,3,3 (4) | 48 |
| N55† | 90 | 4,3,2,1,0 (2) | 90 | 3,2,2,2,1 (2) | 90 | 3,3,3,2,1 (3) | 90 | 3,3,3,2 (3) | 90 |
| N75† | 79 | 3,2,2,1 (2) | 90 | 4,4,3,1,0 (3) | 90 | 5,5,5,4,3 (5) | 78 | 5,5,4,1,1 (4) | 84 |
| N105† | 92 | 5,5,4,4,4 (4) | 90 | 5,4,4,4,3 (4) | 90 | 5,5,4,4,3 (4) | 90 | 4,4,4,4 (4) | 87 |
| N127† | 85 | 5,4,4,0 (4) | 60 | 5,4,4,4,3, (4) | 60 | 5,5,5,4,4 (5) | 57 | 5,5,5,4 (5) | 43 |
| N146* | 77 | 4,3,3,3 (3) | 69 | 2,1,1,1 (1) | 90 | 2,2,2,1 (2) | 65 | 4,3,3,3 (3) | 64 |
| N165† | 87 | 5,5,5,4,4 (5) | 49 | 4,4,4,4,4 (4) | 49 | 5,5,5,4,4 (5) | 41 | 5,5,5,4,4 (5) | 47 |
| N186* | 72.5 | 5,4,4,3 (4) | 65 | 4,4,4,2 (4) | 81 | 5,5,4,4,4 (4) | 66 | 4,4,4,4 (4) | 65 |
| N206† | 73 | 5,4,4,4,4 (4) | 39 | 5,5,5,5 (5) | 38 | 5,5,5,5,4 (5) | 34 | 5,5,5,5,2 (5) | 33 |
| N232* | 71 | 4,4,4,4,2 (4) | 52 | 4,3,2,2,1 (2) | 67 | 4,4,3,3,1 (3) | 62 | 4,4,4,1 (4) | 50 |
| N262* | 71 | 5,5,5,4,4 (5) | 28 | 4,4,4,3 (4) | 32 | 5,5,5,5,4 (5) | 28 | 5,5,4,4,4 (4) | 27 |
| N283* | 75 | 4,4,4,3 (4) | 67 | 4,4,4,4,4 (4) | 68 | 4,4,4,3 (4) | 70 | 5,4,4,4 (4) | 60 |
| N310* | 66 | 5,5,5,4,4 (5) | 31 | 5,5,5,5,4 (5) | 34 | 5,4,4,4,4 (4) | 38 | 5,5,5,4 (5) | 30 |
| N331* | 86 | 5,5,4,4,4 (4) | 44 | 5,5,5,4 (5) | 53 | 5,5,5,5,4 (5) | 46 | 5,5,5,5 (5) | 44 |

*C3H/A$^{vy}$; †CBA/lac.

**Table I** (continued)

(*b*) *Cryopreserved tumour cells:*

| *Tumour* | *No. of viable cells inoculated (viability)* | *Control (median)* | *Trypsin (median)* | *Hyaluronidase (median)* | *Neuraminidase (median)* | *Days at autopsy* |
|---|---|---|---|---|---|---|
| N328* | $0.2 \times 10^6$ (50%) | 3,2,2,2,0 (2) | 1,0,0,0,0 (0) | 2,1,1,0,0 (1) | 2,1,1,0,0 (1) | 92 |
| N383* | $0.3 \times 10^6$ (54%) | 1,1,1,1,0 (1) | 1,1,0,0,0 (0) | 1,1,1,1,0 (1) | 1,1,1,0 (1) | 89 |
| N439* | $0.25 \times 10^6$ (45%) | 3,1,1,1,1 (1) | 1,0,0,0,0 (0) | 2,1,1,1,1 (1) | 2,1,1,0 (1) | 92 |
| N476* | $0.35 \times 10^6$ (48%) | 1,1,1,1,0 (1) | 1,0,0,0 (0) | 1,1,1,1,1 (1) | 1,1,0,0,0 (0) | 93 |
| N477* | $0.3 \times 10^6$ (50%) | 1,1,1,1,1 (1) | 0,0,0,0,0 (0) | 2,2,1,1,1 (1) | 1,1,1,1,0 (1) | 102 |
| E36.1* | $0.4 \times 10^6$ (47%) | 1,1,1,0 (1) | 1,1,0,0,0 (0) | 1,1,1,0 (1) | 1,1,1,0,0 (1) | 99 |
| E37.1* | $0.55 \times 10^6$ (54%) | 2,1,1,1,1 (1) | 1,1,0,0,0 (0) | 2,2,1,1,1 (1) | 2,1,1,1,0 (1) | 87 |
| N641* | $0.4 \times 10^6$ (48%) | 3,3,3,1 (3) | 1,1,1,1,0 (1) | 2,2,1,1,1 (1) | 3,1,1 (2) | 91 |
| P029* | $0.2 \times 10^6$ (36%) | 4,2,2,2 (2) | 4,4,4,4,1 (4) | 4,4,2 (4) | 5,4,4,3,3 (4) | 82 |

*Strain of recipient = C3H/A$^{vy}$.

**Table IIa** Effects of enzymes on inert substrates

| | *Radioactive release (c.p.m.) from $^{125}$I-labelled fibrin Mean ($\pm$s.d.)** | *Release of N-acetyl neuraminic acid from N-acetyl neuraminlactose ($\mu$mol)* | *Release of N-acetyl glucosamine from hyaluronic acid ($\mu$mol)* |
|---|---|---|---|
| Trypsin 0.1% | 14979.6 ($\pm$868.6) | $1.3\times10^{-3}$ | $5.2\times10^{-3}$ |
| Neuraminidase 12.5 u/ml | 191.6 ($\pm$12.2) | $8.6\times10^{-3}$ | $22.9\times10^{-3}$ |
| Hyaluronidase 0.2% | 367 ($\pm$44.1) | $3.0\times10^{-3}$ | $165\times10^{-3}$ |
| MEM | 212 ($\pm$23.1) | $2.7\times10^{-3}$ | — |

*Triplicate tests.

**Table IIb** Release of N-acetylneuraminic acid (NANA) from tumour cell surfaces by neuraminidase

| *Tumour* | *Amount NANA released (means of duplicates, blanks and controls subtracted)* |
|---|---|
| UST 451 | 0.45 $\mu$g $10^{-7}$ cells |
| UST 453 | 0.36 $\mu$g $10^{-7}$ cells |
| UST 457 | 0.40 $\mu$g $10^{-7}$ cells |
| UST 462 | 0.32 $\mu$g $10^{-7}$ cells |
| Mean $\pm$ s.d. = 0.38 $\pm$ 0.06 $\mu$g NANA $10^{-7}$ cells | |

this region confirming that the split products had come from the cells (results not shown). Two-dimensional electrophoresis of Nonidet P-40 extracts of cells treated with neuraminidase showed a spot in the basic region of the gel (Figure 2, arrow) which is not present in the gel of the control (non-digested cells)—this is interpreted as resulting from the splitting of N-acetyl neuraminic acid from a parent glycoprotein, giving rise to a more basic derivative. No differences were seen between 2-d electropherograms of Nonidet P-40 extracts of control and hyaluronidase treated cells nor between gels incubated with $^{125}$I-WGA whether cells were controls, neuraminidase or hyaluronidase treated.

The above studies were repeated on 6 tumours and resulted in similar findings.

The results of the chemical analysis for N-acetyl neuraminic acid (Table IIb) confirmed that there is release of this molecule from the cell surface due to the action of neuraminidase. The release of N-acetyl glucosamine due to the action of hyaluronidase could not be convincingly shown: experimental values were not substantially different from controls.

### Discussion

The observation that trypsin treatment prior to reinoculation did mildly alter the pulmonary colonisation potential of spontaneous mammary tumour cells was sufficiently reproducible (in 14 tumours, the colonisation potential was reduced, and in 4 tumours, increased) to suggest that the protein composition of the cell surface has some influence on metastatic spread. The concomitant finding that neuraminidase treatment, which effectively liberated some N-acetyl neuraminic acid from the cell surface, did not demonstrably affect colonisation potential or distribution of colonies does not necessarily imply that surface sialyl residues do not affect the metastatic process in these tumours. In these particular cells, for instance, determinants of metastatic capability could lie deeper in the cell surface and not accessible to the enzymes under the conditions employed.

Four of the 24 tumours showed an increase in pulmonary colonisation after trypsin treatment. As they were all naturally-occurring tumours (i.e. not derived from a single cell line or transplantable tumour) each could *a priori* have individual variations in cell surface characteristics and hence different responses to enzyme treatment. The essential observation here is that in 18/24 tumours trypsin mediated alteration of cell surface composition resulted in altered pulmonary colonisation potential whereas surface alterations induced by other enzymes had no distinct effect on behaviour.

The differences in viability between the freshly-disaggregated tumour cells and the cryopreserved ones (Tables Ia and b) are unlikely to account for the more frequent observation of trypsin-altered colonisation potential in the latter group of tumours. Extensive studies with cells from these

**Figure 1** Release of polypeptides from tumour cell surfaces by trypsin. Lane 1: control, showing a band due to soybean trypsin inhibitor (SBTI); lane 2; trypsin treated cells, showing the presence of ~8 small polypeptides ranging in mol. wt from 5–18 k. These have been specifically cleaved from the cell surface by trypsin since the same bands cannot be seen in the control lane. Lane 3: mol. wt standards—*E.coli* RNA polymerase subunits (165 k, 155 k, 39 k); bovine serum albumin (68 k), SBTI (21.5 k), cytochrome C (12.5 k), aprotinin (6.5 k) (all from Boehringer). Gel stained with Coomassie blue.

mammary tumours (Tarin and Price, 1979; Price & Tarin, 1982) have demonstrated that the proportion of dead cells has no effect on pulmonary colonisation, providing the numbers of viable cells inoculated is constant. This precaution was taken with the current work. Hence we infer that the difference between the 60% (9/15 tumours) incidence of trypsin-altered tumour behaviour in the freshly-disaggregated tumours and the 100% change in the cryopreserved group is attributable to sampling variation.

The distribution of deposits was unaffected, even in the groups receiving trypsin-treated cells, where an effect on *degree* of colonisation was noted. This seems to be at variance with the findings of earlier investigations (Sinha & Goldenberg, 1974; Weiss *et al.*, 1974), in which it was found that enzyme treatment of the surfaces of various serially-transplanted tumour cell lines and of lymphocytes (Woodruff & Gesner, 1969; Woodruff, 1974) resulted in altered distribution of inoculated cells. However, these previous studies involved very short-term observations, the longest continuing for only 3 days after inoculation, and most used cytoplasmic labels, such as $^{51}Cr$ which can be reutilised by host cells. Weiss (1980), using $^{125}IUdR$, a non-reutilised nuclear label, did not find any redistribution of Walker/256 tumour cells to the liver after neuraminidase treatment, as suggested by the other work cited above including his own earlier work with different tumour cell types (Weiss *et al.*, 1974).

We have no direct knowledge of whether the organ distribution of *cells* from these spontaneous mammary carcinomas was altered by the enzyme treatment. However, we know from other experiments (Tarin & Price, 1981) that the distribution of eventual *deposits* is influenced by whether the cells arresting in a site can grow there. Although cells of these mammary tumours will grow in some extrapulmonary sites when inoculated directly via the aorta (Juacaba *et al.*, in press), good evidence has been provided by Weiss (1980) that cells induced to pass through the lung into the arterial blood instead of arresting there, are "processed" by this organ and become less capable of producing tumour deposits in organs downstream. Thus, the absence of any alteration in distribution of tumour *deposits* in this investigation is not necessarily incompatible with the earlier short-term studies on cell distribution.

While enzyme modification of cells from these spontaneous mammary tumours had no *striking* effect on the degree or distribution of deposits after i.v. inoculation, there remains considerable circumstantial evidence (see above) that the constitution of the cell surface is an important determinant of the degree and distribution of

**Figure 2** Coomassie blue stained electropherograms of Nonidet-P40 extracts of control (a), hyaluronidase (b) and neuraminidase (c) treated tumour cell surfaces of UST 453. The acidic end of each gel is at the left. The basic derivative formed from the neuraminidase treated cells is arrowed. This derivative is probably formed by loss of sialic acid from a glycoprotein due to the action of neuraminidase, thus rendering the derivative relatively more basic than its parent molecule. There is no detectable protein in this region of the control gel.

metastatic colonisation. The interesting report from Poste & Nicolson (1980), that they were able to increase the colonising ability of B16/F1 (low-colonising cells) by fusing into them membrane vesicles budded off from B16/F10 (high-colonising cells), directly supports this view. F1 vesicles fused into F10 cells did not reduce the colonisation potential of the latter. The authors suggested that the surface components responsible for localisation of F10 cells in the lungs were not effectively diluted out by the addition of the F1 vesicles. Similarly with our cells, enzyme action, demonstrated by the assays and PAGE, did remove *some* cell surface components (see also Ceriani *et al.*, 1978) but the removal of more may be required for a greater difference in the final degree of lung colonisation by the treated tumour cells to be detected.

The finding of a modest effect of enzyme modification on colonisation potential in our experiment should also be considered in the light of some of our recent dose-response studies. It was found that the pulmonary colonisation potential of a given tumour, whether high or low, is stable even when the dose is reduced to one-twentieth of that used in the experiments reported here (Price *et al.*, 1982). Hence, the change in numbers of cells lodging and growing in the lung as a result of surface modifications would have had to be quite large to be detectable.

Further factors needing to be taken into account in the interpretation of our results are the ability of cells to repair membrane damage and that turnover of membrane components is rapid, some 20–30% being reconstituted within 2h at 37°C and the remainder by 24h (Kaplan *et al.*, 1979; Davies & Trotter, 1981). Although in our experiments cells were kept chilled after enzyme treatment and were rapidly distributed once inside the animal (Potter *et al.*, in press), factors such as membrane reconstitution prior to inoculation, and even after arrest, could tend to reduce the effects of the experimental interference.

These considerations demonstrate that the results obtained, although not dramatic, justify further experiments on the role of membrane constituents in metastatic spread by direct modification, replacement or transplantation of membrane constituents.

This work was supported by a grant from the Medical Research Council of Great Britain, whose support is gratefully acknowledged. We also wish to thank Mr. K. Millican and Mr. N. Sanders of the Oxford University Medical School Animal House for animal care, and Mr. R. Holton for photography. We particularly appreciate the help of Mrs. P. Messer in coordination of this work and in preparation of the manuscript.

### References

AMINOFF, D. (1961). Methods for the quantitative estimation of N-acetylneuraminic acid and their application to hydrolysates of sialomucoids. *Biochem. J.*, **81**, 384.

BOSMANN, H.B., BIEBER, G.F., BROWN, A.E. & 4 others (1973). Biochemical parameters correlated with tumour cell implantation. *Nature*, **246**, 487.

BRAMWELL, M.E. & HARRIS, H. (1978). An abnormal membrane glycoprotein associated with malignancy in a wide range of different tumours. *Proc. Roy. Soc.* (London B,) **201,** 87.

BRUNSON, K.W., BEATTIE, G. & NICOLSON, G.L. (1978). Selection and altered properties of brain-colonizing metastatic melanoma. *Nature,* **272,** 543.

CERIANI, R.L., PETERSON, J.A. & ABRAHAM, S. (1978). The removal of cell surface materials by enzymes used to dissociate mammary gland cells. *In vitro,* **14,** 887.

CHATTERJEE, S.K. & KIM, U. (1977). Galactosyltransferase activity in metastasizing and nonmetastasizing rat mammary carcinomas and its possible relationship with tumour cell surface antigen shedding. *J. Natl Cancer Inst.,* **58,** 273.

DAVIS, H.W. & TROTTER, M.D. (1981). Synthesis and turnover of membrane glycoconjugates in monolayer culture of pig and human epidermal skin. *Br. J. Dermatol,* **104,** 649.

FIDLER, I.J. (1973). Selection of successive tumour lines for metastasis. *Nature* (New Biol.) **242,** 148.

FIDLER, I.J. (1978*a*). Tumour heterogeneity and the biology of cancer invasion and metastasis. *Cancer Res.,* **38,** 2651.

FIDLER, I.J. (1978*b*). General consideration for studies of experimental cancer metastasis. *Methods Cancer Res.,* **15,** 399.

GESNER, B.M. & GINSBURG, V. (1964). Effect of glycosidases on the fate of transfused lymphocytes. *Proc. Natl Acad. Sci.,* **52,** 750.

JUACABA, S.F., JONES, L.D. & TARIN, D. (1983). Organ preferences in metastatic colony formation by spontaneous mammary carcinomas after intra-arterial inoculation. *Invasion Metastasis* (in press).

KAPLAN, G., UNKELESS, J.C. & COHN, Z.A. (1979). Insertion and turnover of macrophage plasma membrane proteins. *Proc. Natl Acad. Sci.,* **76,** 3824.

KIM, U., BAUMLER, A., CARRUTHERS, C. & BIELAT, K. (1975). Immunological escape mechanism in spontaneously metastasizing mammary tumours. *Proc. Natl Acad. Sci.,* **72,** 1012.

LASKEY, R.A. & MILLS, A.D. (1977). Enhanced autoradiographic detection of $^{32}P$ and $^{125}I$ using intensifying screens and hypersensitized film. *FEBS Lett.,* **82,** 314.

NICOLSON, G.L. (1974). The interactions of lectins with animal cell surfaces. *Int. Rev. Cytol.,* **39,** 89.

POSTE, G. & NICOLSON, G.L. (1980). Arrest and metastasis of blood-borne tumour cells are modified by fusion of plasma membrane vesicles from highly metastatic cells. *Proc. Natl Acad. Sci.,* **77,** 399.

POTTER, K.M., JUACABA, S.F., PRICE, J.E. & TARIN, D. (1983). Observations on organ distribution of fluorescein labelled tumour cells released intravascularly. *Invasion Metastasis* (in press).

PRICE, J.E. & TARIN, D. (1981). Lectin agglutinability of mammary tumours with differing metastatic colonisation potentials. *Differentiation,* **20,** 264.

PRICE, J.E., CARR, D., JONES. L.D., MESSER, P. & TARIN, D. (1982). Experimental analysis of factors affecting metastic spread using naturally-occurring tumours. *Invasion Metastasis,* **2,** 77.

PRICE, J.E & TARIN, D. (1982). Retention of "metastatic" colonisation potential by cells of spontaneous primary tumours after cryopreservation. *Br. J. Cancer,* **45,** 790.

REISSIG, J.L., STROMINGER, J.L. & LELOIR, L.F. (1955). A modified colourimetric method for the estimation of N-acetylamino sugars. *J. Biol. Chem.,* **217,** 959.

RIEBER, M. & RIEBER, M.S. (1981). Metastatic potential correlates with cell-surface protein alterations in B16 melanoma variants. *Nature,* **293,** 74.

SCHLESINGER, M. & ISRAEL, E. (1974). The effect of lectins on the migration of lymphocytes *in vivo. Cell. Immunol.,* **14,** 66.

SINHA, B.K. & GOLDENBERG, G.J. (1974). The effect of trypsin and neuraminidase on the circulation and organ distribution of tumor cells. *Cancer,* **34,** 1956.

TARIN, D. & PRICE, J.E. (1979). Metastaic colonization potential of primary tumour cells in mice. *Br. J. Cancer,* **39,** 740.

TARIN, D. & PRICE, J.E. (1981). Influence of microenvironment and vascular anatomy on "metastatic" colonization potential of mammary tumors. *Cancer Res.,* **41,** 3604.

TARIN, D., HOYT, B.J. & EVANS, D.J. (1982). Correlation of collagenase secretion with metastatic-colonization potential in naturally-occurring murine mammary tumours. *Br. J. Cancer,* **46,** 266.

WARREN, L. (1959). The thiobarbituric acid assay of sialic acids. *J. Biol. Chem.,* **234,** 1971.

WEISS, L. (1980). Cancer cell traffic from the lungs to the liver: an example of metastatic inefficiency. *Int. J. Cancer,* **25,** 385.

WEISS, L., GLAVES, D. & WAITE, D.A. (1974). The influence of host immunity on the arrest of circulating cancer cells, and its modification by neuraminidase. *Int. J. Cancer,* **13,** 850.

WOODRUFF, J.J. (1974). Role of lymphocyte surface determinants in lymph node homing. *Cell. Immunol.,* **13,** 378.

WOODRUFF, J.J. & GESNER, B.M. (1968). Lymphocytes: circulation altered by trypsin. *Science,* **161,** 176.

WOODRUFF, J.J. & GESNER, B.M. (1969). The effect of neuraminidase on the fate of transfused lymphocytes. *J. Exp. Med.,* **129,** 551.